# Widespread skin sclerosis with impaired mobility

**DOI:** 10.1016/j.jdcr.2025.10.017

**Published:** 2025-10-28

**Authors:** Zhong-Zhou Huang, Jian-Chi Ma, Ping Huang, Ze-Hai Wang, Qing Guo, Liang-Chun Wang

**Affiliations:** a Department of Dermatology, Sun Yat-sen Memorial Hospital, Sun Yat-sen University, Guangzhou, China; b Department of Epidemiology and Public Health, School of Public Health, Sun Yat-sen University, Guangzhou, China

**Keywords:** alopecia, connective tissue disease, disabling pansclerotic morphea (DPM), sclerosis

## Case description

**Fig 1:**
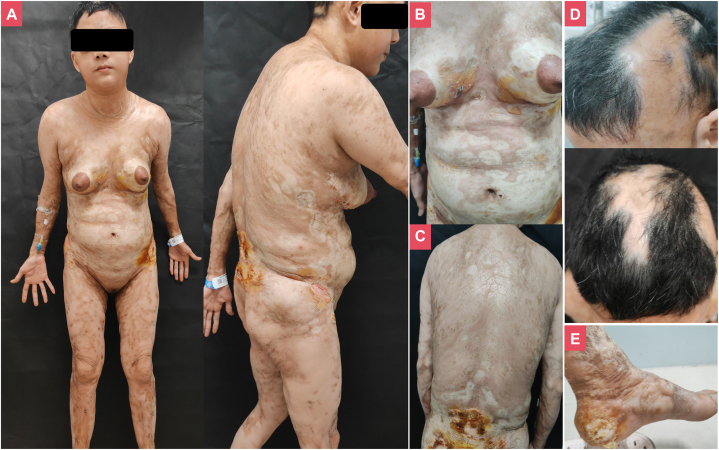
Disabling pansclerotic morphea. **A,** Front view and lateral view; **(B)** Bulla on the chest; **(C)** Back; **(D)** Diffuse alopecia; **(E)** Ulcer on foot heel.

**Fig 2:**
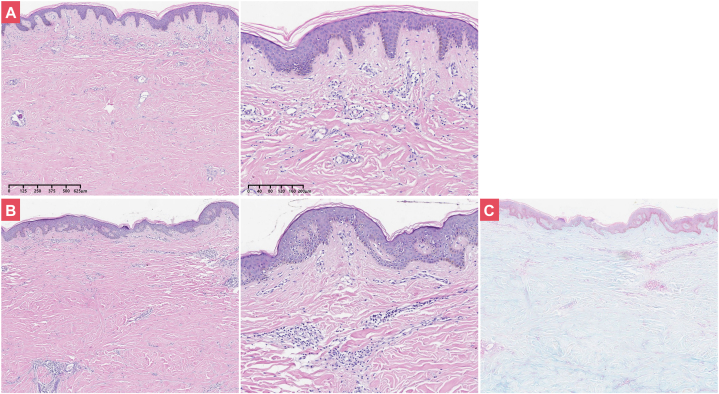
Histopathologic examination of skin lesions. **A,** Chest lesion (left: ×40, right: ×100); **(B)** Back lesion (left: ×40, right: ×100); **(C)** Negative Alcian blue staining for back lesion. Note: the dermis was thickened and replaced by homogeneous, sclerotic collagen, while perivascular lymphocytic infiltration around dermal blood vessels.

A 45-year-old woman presented with recurrent facial edema with a positive antinuclear antibody titer of 1:80, diagnosed with undifferentiated connective tissue disease at a hospital 3 years ago, receiving methylprednisolone (4 mg once daily) and cyclosporine (50 mg twice daily), but the therapeutic effect was poor. Over the past year, she developed red plaques on her trunk and limbs that progressed to sclerosis, along with rapidly progressing alopecia (Fig 1). Physical examination revealed diffuse cutaneous sclerosis with ulcerations (chest, back, buttocks, and heels) and purulent discharge, limited movement of the upper and lower extremities. Laboratory tests showed antinuclear antibody 1:80, but were negative for ENA (including anti-Scl-70), pemphigus antibodies and KL-6, without evidence of visceral fibrosis involvement (chest CT, cardiac and renal ultrasound). Skin biopsies from the chest and back revealed dermal fibroplasia with inflammatory cell infiltration, with negative for Alcian-blue staining, findings consistent with morphea (Fig 2).

**Question: What is the most likely diagnosis for this patient?**

A. Morphea
B. Undifferentiated connective tissue disease
C. Scleromyxedema
D. Systemic sclerosis
E. Disabling pansclerotic morphea

## Answer discussion

The correct answer is **E,** the disabling pansclerotic morphea (DPM).

Morphea is a chronic autoimmune disorder marked by progressive cutaneous sclerosis that can inflict functional impairment and irreversible disfigurement, while severe subtypes and refractory cases mandate escalation to systemic immunosuppressive or immunomodulatory regimens.^1^ The present case was a typical variant with significant functional impairment and irreversible disfigurement. DPM is the most severe, rarest end of the localized scleroderma spectrum - a relentless, system-wide inflammatory disease.^2^ As to this case, she experienced a 3-year history of connective tissue disease with 1 year of disease exacerbation and age 45 years, which was similar to a previous investigation on keloidal scleroderma.^3^ To date, no clear clinical or laboratory criteria have been established for DPM; however, it is unequivocally characterized as a disabling, pansclerotic disorder that spares the internal viscera from fibrosis.

DPM usually lacks overt abnormal immunologic markers. In this case, the skin biopsies revealed thickening and homogenization of collagen fibers in the deep dermis and subcutis (Fig 2), while antinuclear antibody was only 1:80 and all other laboratory parameters were within normal limits. Compared with this case, the patient with refractory morphea presented with infiltrated plaques on both legs and her laboratory tests including antibody panels were negative, while the skin condition was significantly improved with the use of upadacitinib (a JAK 1-inhibitor), suggesting that refractory morphea is associated with many immune-mediated disorders.^4^ In the current case, treatment was initiated with methylprednisolone (8 mg once daily) and methotrexate (10 mg QW). Four days later, the inflammatory lesion continued to gradually expand. Subsequently, tofacitinib (5 mg BID) and thalidomide (50 mg QN) were added, leading to resolution of the acute lesions. The use of methylprednisolone and immunomodulators (tofacitinib, methotrexate and thalidomide) improved the severe status. Individual immune dysfunction and environmental changes are key triggers of this kind of disease.^5^ Therefore, exploring individual immune-related genes, epigenetic mechanisms, and environmental factors represents crucial directions for future research.

## Conflicts of interest

None disclosed.
